# Establishment of a Novel Detection Platform for Clostridioides difficile Toxin Genes Based on Orthogonal CRISPR

**DOI:** 10.1128/spectrum.01886-23

**Published:** 2023-06-28

**Authors:** Tong Jiang, Xinyi Hu, Jilu Shen

**Affiliations:** a The First Affiliated Hospital of Anhui Medical University, Hefei, Anhui, China; b Anhui Public Health Clinical Center, Hefei, Anhui, China; University of Manitoba

**Keywords:** *Clostridioides difficile*, orthogonal CRISPR system, immunochromatographic test strips, multiple RPA

## Abstract

Clostridioides difficile is one of the leading pathogens causing nosocomial infection. The infection can range from mild to severe, and rapid identification is pivotal for early clinical diagnosis and appropriate treatment. Here, a genetic testing platform for toxins, referred to as OC-MAB (orthogonal CRISPR system combined with multiple recombinase polymerase amplification [RPA]), was developed to detect the C. difficile toxin genes *tcdA* and *tcdB*. While recognizing the amplified products of the *tcdA* gene and the *tcdB* gene, Cas13a and Cas12a could activate their cleavage activities to cut labeled RNA and DNA probes, respectively. The cleaved products were subsequently identified by dual-channel fluorescence using a quantitative PCR (qPCR) instrument. Finally, they could also be combined with labeled antibodies on immunochromatographic test strips to achieve visual detection. The OC-MAB platform exhibited ultrahigh sensitivity in detecting the *tcdA* and *tcdB* genes at levels of as low as 10^2^ to 10^1^ copies/mL. When testing 72 clinical stool samples, the sensitivity (95% confidence interval [CI], 0.90, 1) and specificity (95% CI, 0.84, 1) of the single-tube method based on the fluorescence readout was 100%, with a positive predictive value (PPA) value of 100% (95% CI, 0.90, 1) and a negative predictive value (NPA) value of 100% (95% CI, 0.84, 1), compared to the results of qPCR. Likewise, the sensitivity of the 2-step method based on the test strip readout was 100% (95% CI, 0.90, 1), while the specificity was 96.3% (95% CI, 0.79, 0.99), with a PPA of 98% (95% CI, 0.87, 0.99) and an NPA of 100% (95% CI, 0.90, 1). In short, orthogonal CRISPR technology is a promising tool for the detection of C. difficile toxin genes.

**IMPORTANCE** C. difficile is currently the primary causative agent of hospital-acquired antibiotic-induced diarrhea, and timely and accurate diagnosis is crucial for hospital-acquired infection control and epidemiological investigation. Here, a new method for the identification of C. difficile was developed based on the recently popular CRISPR technology, and an orthogonal CRISPR dual system was utilized for the simultaneous detection of toxin genes A and B. It also uses a currently rare CRISPR dual-target lateral flow strip with powerful color-changing capabilities, which is appropriate for point-of-care testing (POCT).

## INTRODUCTION

Nucleic acid detection is a major molecular diagnostic application with significant development and advancements over the past few decades. In particular, quantitative PCR (qPCR) technology has become the gold standard for the detection of various pathogenic bacteria in recent years. However, this method depends on a complex laboratory environment and expensive experimental equipment, limiting its use in resource-scarce environments (1–4). Therefore, it is crucial to establish a portable, rapid, highly sensitive, and specific nucleic acid detection technology for controlling pathogen infection (1).

CRISPR/Cas (clustered regularly interspaced short palindromic repeat/CRISPR associated) is the adaptive immune system of bacteria or archaea. Recently, this immune system has been widely used in molecular biology and has become one of the most powerful platforms for gene editing, known as the “molecular magic sword” (5–7). In particular, the recent discovery of lytic activity has created new opportunities and expanded the possibilities for molecular diagnostics. In 2015, Zetsche et al. discovered that Cas12a is a single RNA-guided endonuclease that targeted double-stranded DNA (dsDNA) (*cis*-cleavage activity) (8). Subsequently, Chen et al. found that Cas12a also exhibited nonspecific single-stranded DNA (ssDNA) cleavage (*trans*-lysis activity) after the cleavage of double-stranded DNA (9). Based on this principle, Cas12a *trans*-lysis activity was combined with recombinase polymerase amplification (RPA) to develop the DETECTR (DNA endonuclease-targeted CRISPR *trans*-reporter) nucleic acid detection platform for detecting different human papillomavirus (HPV) types (9). Li et al. developed a time-efficient and low-cost nucleic acid detection technology by combining PCR technology with Cas12a, known as HOLMES (1-h low-cost multipurpose highly efficient system) (10). Similarly, the Cas13a protein (formerly known as C2C2) has *trans*-cleavage activity and can be used for specific nucleic acid detection (11, 12). For example, the famous SHERLOCK (specific high-sensitivity enzymatic reporter unlocking) platform was established (13). The development of these new biosensing platforms for nucleic acid detection based on the CRISPR/Cas system has significantly advanced the field of nucleic acid detection and presented new opportunities for point-of-care testing (POCT). So far, nucleic acid detection methods based on CRISPR technology have been successfully used for various pathogens, including severe acute respiratory syndrome coronavirus 2 (SARS-CoV-2) (14–19), African swine fever virus (ASFV) (20, 21), plant viruses (22), and foodborne pathogens (23).

In a previous study, we used multiplex RPA technology to amplify the Clostridioides difficile toxin genes A and B. Later, we transferred the amplicons to the Cas12a system for individual cleavage. This platform successfully detected C. difficile toxin genes using CRISPR technology (24). However, although the development of multiplex RPA can amplify double genes concurrently, the nonspecific cleavage activity of the CRISPR system makes it impossible to perform multiplex detection in one tube. At present, there have been few reports on multiplex diagnoses using CRISPR. Gootenberg et al. proposed the second version of SHERLOCK (the SHERLOCKv2 detection platform) (25). The SHERLOCKv2 detection platform used the four-channel single reaction of orthogonal CRISPR for the first time to detect four targets concurrently, thus realizing multiplex detection using the CRISPR system (25). Subsequently, Tian et al. used orthogonal CRISPR enzymes to establish a single-tube multiplex detection platform for SARS-CoV-2 and ASFV (26). They also developed a portable handheld fluorescence detection device to detect SARS-CoV-2 and ASFV in a single tube (26). Although the orthogonal CRISPR/Cas strategy has been used in many studies (27, 28), including the SHERLOCKv2 platform and the platform established by Tian et al., both of these methods require specialized equipment to interpret the fluorescence outcomes, leading to increased costs.

**FIG 1:**
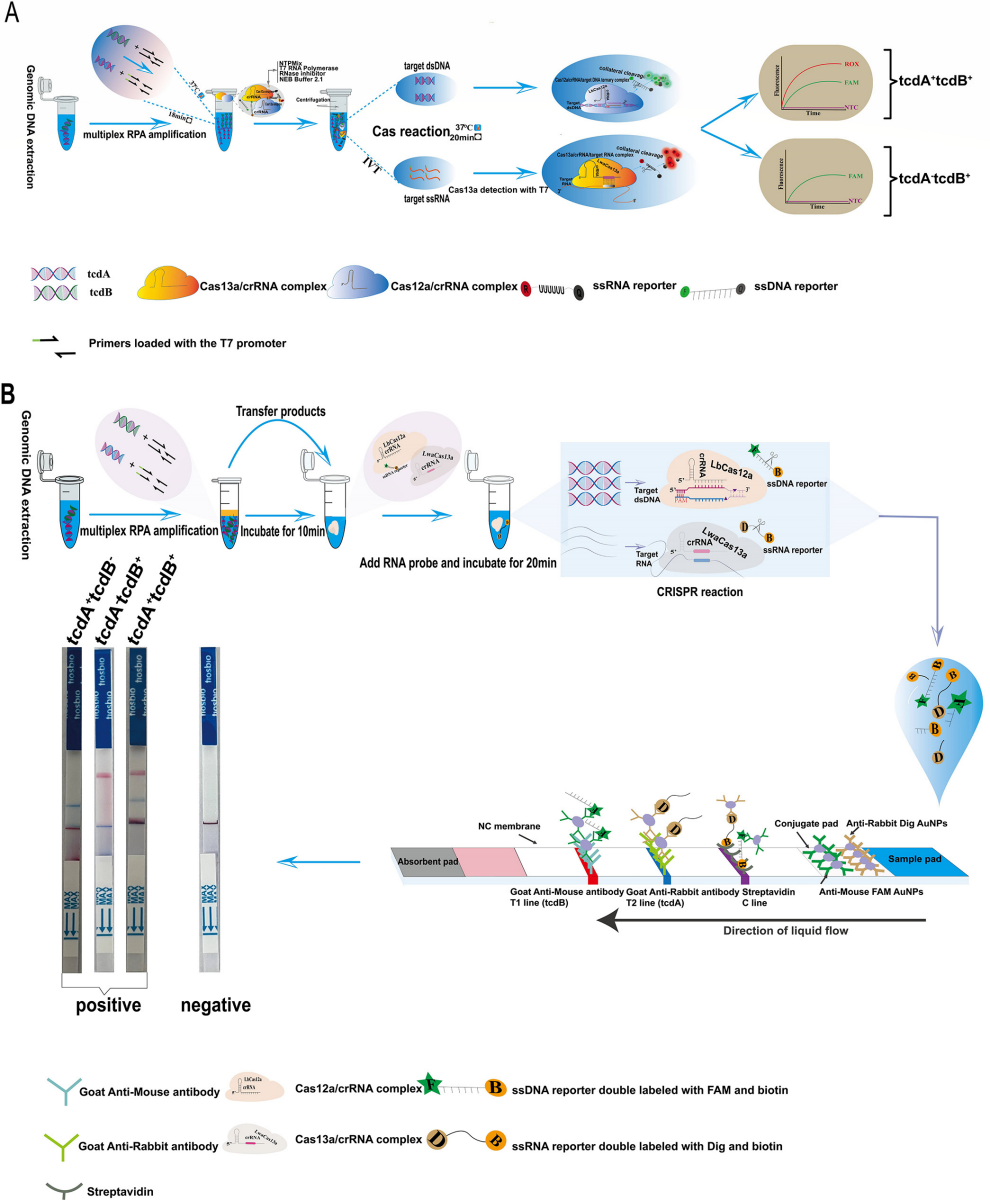
Workflow of the OC-MAB platform. (A) Workflow based on fluorescence. Isothermal amplification combined with CRISPR dual-system cleavage was performed in a single tube, and the results were read out by fluorescence. NTC, no-template control. (B) Workflow based on an immunochromatographic lateral flow strip. A CRISPR dual-system test strip with 2 detection lines was used to detect the products after double-enzyme digestion; the results show a discoloration effect.

This study used an orthogonal CRISPR system to establish a C. difficile toxin gene detection platform known as OC-MAB (orthogonal CRISPR system combined with multiple RPA) (Fig. 1). A novel approach called “CRISPR orthogonal detection” was developed, which involves the use of two kinds of CRISPR/Cas proteins to detect DNA and RNA simultaneously. To the best of our knowledge, no reports of this CRISPR orthogonal detection for C. difficile toxin gene detection are available. Briefly, T7 RNA polymerase transcribes one of the genes into RNA, while the other gene remained unchanged. Next, two genes in a single tube are concurrently detected using the single-stranded DNA cleavage ability of Cas12a and the single-stranded RNA (ssRNA) cleavage ability of Cas13a. Furthermore, we provide two ways to obtain results: the fluorescence and immunochromatographic lateral flow strip methods. We used the dual-CRISPR system test strip for the lateral flow strip method, which allows the simultaneous identification of two genes on the test strip.

## RESULTS

### Integration of multiple RPA and the orthogonal CRISPR system to establish the OC-MAB platform.

To develop a sensitive assay to identify C. difficile, multiple RPA and CRISPR dual systems were integrated to design the OC-MAB platform. The OC-MAB platform detection process is illustrated in Fig. 1. The platform integrated (i) preamplification of C. difficile DNA samples and *in vitro* transcription of T7 (containing a primer labeled with the T7 promoter sequence), (ii) sequence-specific recognition and nonspecific cleavage of Cas12a-CRISPR RNA (crRNA) and Cas13a-crRNA, and (iii) dual-gene visualization of fluorescence and test strip readouts. Briefly, the Cas12a protein, guided by *tcdB*-crRNA, recognized the protospacer-adjacent motif (PAM) site and bound to the target DNA. This activation triggered *trans*-cleavage activity, which cleaved the nearby 6-carboxyfluorescein (FAM)- and black hole quencher 1 (BHQ1)-labeled ssDNA probes or FAM- and biotin-conjugated ssDNA probes. Regarding ssRNA cleavage by Cas13a, the T7 promoter sequence was added to one end of the *tcdA* gene primer. Moreover, the T7 RNA polymerase was included in the mixture, which transcribed the dsDNA amplicon into ssRNA. On the other hand, Cas13a, guided by *tcdA*-crRNA, recognized ssRNA and cleaved carboxy-x-rhodamine(ROX)- and BHQ2-labeled ssRNA probes or digoxigenin (DIG)- and biotin-conjugated ssRNA probes. The fluorescence signal could be detected using a qPCR instrument or other optical instruments with dual channels (Fig. 1A). This platform could also bind to the labeled antibody on the immunochromatographic test strip to exhibit color-coded bands for visual detection (Fig. 1B).

### Verification of the dual system.

**FIG 2:**
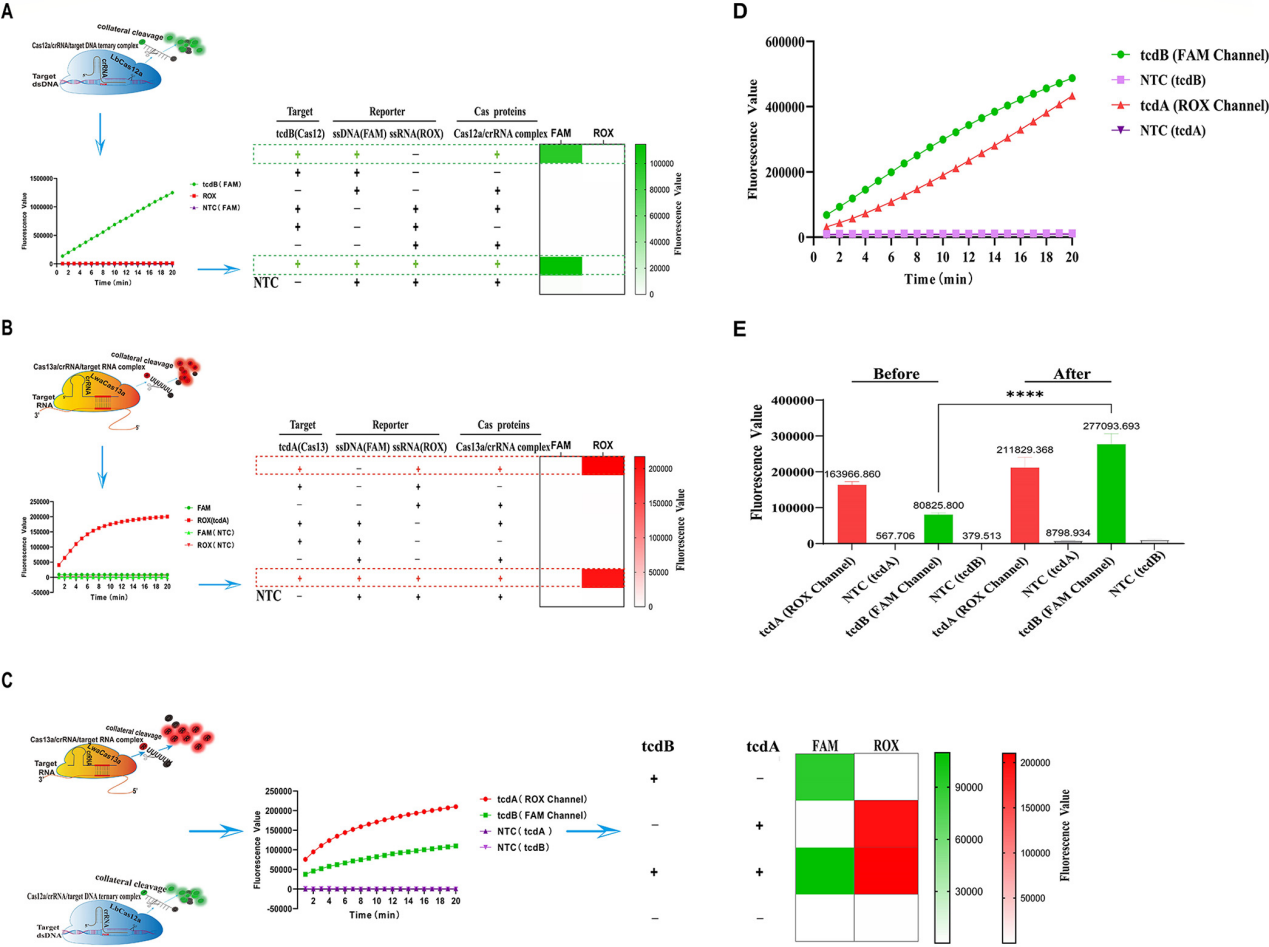
Verification of the cleavage preference of the Cas protein and establishment of the dual-cleavage system. (A) After binding to the target RNA, Cas13a cleaved only the ssRNA reporter. NTC, no-template control. (B) After binding to the dsDNA target, Cas12a cleaved only the ssDNA reporter. NTC, no-template control. (C) Orthogonal detection of the *tcdA* and *tcdB* genes with Cas12a and Cas13a. NTC, no-template control. (D) The CRISPR fluorescence dual system established after optimization. NTC, no-template negative control. (E) Comparison of the fluorescence intensity of the optimized dual system with that of the preoptimized system. ****, *P* < 0.0001. NTC, no-template negative control.

First, to verify the cleavage activity of the Leptotrichia wadei Cas13a(LwaCas13a) protein and the feasibility of the designed LwaCas13a-crRNA, the activity of the LwaCas13a protein was determined using synthetic *tcdA*-RNA as a template. Our results revealed that the Cas13a protein could cleave the target RNA, thereby displaying a strong fluorescence signal (see Fig. S1A in the supplemental material). Besides, the Cas13a protein also demonstrated a robust cleavage ability when the target was replaced with the amplicon generated by RPA (Fig. S1B). These results indicated that the purchased Cas13a protein can be used to detect the *tcdA* gene. Next, in order to successfully establish the single-tube dual system, the Cas13a and Cas12a single-enzyme cleavage systems were explored for the *tcdA* gene and the *tcdB* gene, respectively. To exclude the influence of relevant factors, such as the volume of a single tube and the excess target product after single-tube amplification, on the cleavage efficiency, the lyophilized enzyme component of RPA was mixed and dissolved in a rehydration solution and enzyme-free water beforehand. Following the addition of the primers and the template DNA, 10 μL of the mixture was pipetted, and magnesium acetate (MgOAc) was added, followed by isothermal amplification, which has been verified to have a superior Cas cleavage efficiency in a single tube (Fig. S1C and D). Following this, Cas12a and Cas13a reagents were added to the tube cap, and the single-tube Cas12a system and Cas13a system were successfully established (Fig. S1E and F). Finally, a Cas protein cleavage preference verification test was performed in order to verify the impact of the coexistence of Cas12a and Cas13a on their nuclease activities. To achieve this, ROX- and BHQ2-labeled ssRNA reporter probes were introduced into the Cas12a single-enzyme-digestion system. After amplification, the fluorescence signals of the FAM and ROX channels were monitored at 37°C using a qPCR instrument. Our results revealed that only ssDNA probes were cleaved (Fig. 2A and Fig. S2A). Similarly, in the Cas13a single-digestion system, FAM and BHQ1 doubly labeled ssDNA reporter probes were introduced, and the results uncovered that only ssRNA probes were cleaved (Fig. 2B and Fig. S2B). In summary, these results demonstrated that the Cas12a and Cas13a systems showed favorable orthogonal collateral cleavage of ssDNA and ssRNA reporter genes without interfering with each other in the presence of the corresponding targets.

### Establishment and optimization of the single-tube orthogonal CRISPR dual system.

After the validation of the substrate cleavage preference, a single-tube orthogonal CRISPR dual system was generated based on the fluorescence method (Fig. 2C). Notably, in several repeated experiments, when the components of the two systems were mixed, the cleavage efficiency of the Cas12a protein was relatively low, resulting in a low fluorescence curve. Therefore, to enable a high cutting efficiency of the single-tube CRISPR dual system, the primary parameters were optimized. The first step to accomplish this was to determine the ideal concentration ratio of Cas12a to crRNA. Thus, a series of different concentration ratios was set, and the results showed that the fluorescence effect was optimal when the concentrations of both Cas12a and crRNA were 200 nM (Fig. S2C). The concentrations of Cas13a and the corresponding crRNA were used according to the manufacturer’s instructions. After this, the concentration of T7 RNA polymerase was optimized, and the results showed that the fluorescence effect was optimal at a concentration of 5 U/μL (Fig. S2D). Finally, the NTP Mix concentration was optimized, and the results showed that the best results were obtained at a concentration of 5 mM (Fig. S2E). After optimization, the final fluorescence single-tube CRISPR dual system included Cas12a (200 nM), Cas12a-crRNA (200 nM), Cas13a (50 nM), Cas13a-crRNA (120 nM), T7 RNA polymerase (5 U/μL), NTP Mix (5 mM), a murine RNase inhibitor, a FAM-CCCCC-BHQ1 ssDNA probe (1 μM), a ROX-UUUUU-BHQ2 ssRNA probe (5 μM), and 10× NEBuffer 2.1. Figure 2D and E depict that the optimized CRISPR dual system exerts a higher cleavage efficiency.

### Establishment of a high-efficiency CRISPR orthogonal dual system based on immunochromatography.

In order to enable a more diversified readout of the results from the OC-MAB platform, a CRISPR dual-system-specific test strip was constructed. Figure 1B illustrates the working principle of the test strip. The CRISPR dual-system lateral flow strip consisted of a sample pad, a conjugate pad, a nitrocellulose filter membrane (NC membrane), an absorbent pad, a bottom plastic pad, detection line 1 (T1), detection line 2 (T2), and a control line (C line). In short, the coupling pad was precoated with anti-mouse FAM gold nanoparticles (AuNPs) and anti-rabbit DIG AuNPs, the control line was coated with streptavidin, T1 was coated with goat anti-mouse secondary antibody, and T2 was coated with goat anti-rabbit secondary antibody. When the doubly digested product entered the sample pad and flowed upward to the coupling pad, it was bound to the above-described antibody-coupled AuNP complex and progressively flowed upward. Upon reaching the control line, the biotin-labeled end of the reporter in the negative samples (the dual system did not possess *trans*-cleavage activity, and the reporter remained unaltered) was captured by streptavidin on the control line, which developed color owing to the accumulation of AuNPs. Concerning positive samples (dual-system *trans*-lysis), the samples gradually flowed upward and reached detection line 2, whereby the cleaved ssDNA substrate conjugated with FAM and AuNPs was captured by goat anti-mouse secondary antibody, showing *tcdB* gene positivity. When the samples reached detection line 1, the goat anti-rabbit secondary captured the cleaved ssRNA substrate conjugated with DIG and was then stably bound to the T1 line, exhibiting *tcdA* gene positivity. Finally, distinct color bands were visible due to the accumulation of AuNPs.

In order to ensure that the negative control developed color only on the C line, the probe concentration was first optimized. Multiple concentrations of the probes were tested, while equimolar concentrations of the two probes were mixed. When the final concentration of the two probes reached 100 nM, only the C line emerged on the strip (Fig. S3A). Interestingly, the lateral flow strip had a color-changing function. In the water-probe group, the T2 line of the test strip turned blue when only FAM-labeled probes were added, while the quality control line turned red. If only a DIG-labeled probe was added, the T1 line turned red (Fig. S3B). Next, the efficiency of the single-enzyme digestion of test strips was explored at different concentrations. When the system contained only Cas12a reaction components and DIG-conjugated RNA probes, the concentration of Cas12a was increased to 5 μM. Additionally, the T1 line had the darkest color, whereas the other systems were colorless or of low efficiency (Fig. S3C). Meanwhile, the cleavage efficiency was examined under different buffer conditions, and our results showed that commercially available buffers did not significantly impact the efficiency of Cas12a single-enzyme digestion. When an additional 1 μL of Mg^2+^ was added to NEBuffer 2.1, the intensity of the T1 line was significantly increased (Fig. S3C). Similarly, no color developed in the T2 line when the system contained only Cas13a and FAM-conjugated DNA probes. In comparison, increasing the concentration of *tcdA*-crRNA to 10 μM resulted in the formation of blue bands, while increasing the concentration of Cas13a to 5 μM did not alter the intensity of the bands (Fig. S3D). Similar to the Cas12a system, the addition of 1 μL of Mg^2+^ to NEBuffer 2.1 significantly increased the intensity of the T2 line (Fig. S3D). Collectively, these results signified that the addition of a small amount of Mg^2+^ to the system enhanced the cleavage efficiency of Cas proteins.

Next, single-enzyme digestion systems were integrated while the CRISPR orthogonal dual system was established using immunochromatography. However, the result was not as satisfactory as anticipated, and the color of the T2 lines changed, whereas that of the T1 lines did not (Fig. S3E). We speculated that the high concentration of the NTP Mix solution might inhibit the cleavage of Cas12a. To validate this hypothesis, the concentration of the NTP Mix solution was reduced to 10 mM. Both detection lines were colored at this level, but the performance was not particularly outstanding (Fig. S3E). Subsequently, the approach was modified for sample addition. Cas12a, Cas13a, two crRNAs, a FAM-7TA7T-biotin probe, NTP Mix (10 mM), T7 RNA polymerase (50 U/μL), and the amplified product were added to the PCR tube, and the mixture was incubated at 37°C for 10 min. In this step, Cas12a cleavage and T7 transcription cooccurred *in vitro*. Next, a DIG-12U-biotin probe, an RNase inhibitor, Mg^2+^, and water were added to the system. The mixture was subsequently incubated at 37°C for 20 min, during which Cas13a was cleaved. After the process, strong bands were visualized on both test lines (Fig. S3E). Overall, an efficient CRISPR dual-cleavage system that displayed strong color bands on CRISPR dual-system test strips was established through these optimizations (Fig. S4 and Table S3).

### Evaluation of the sensitivity and specificity of the OC-MAB platform.

**FIG 3:**
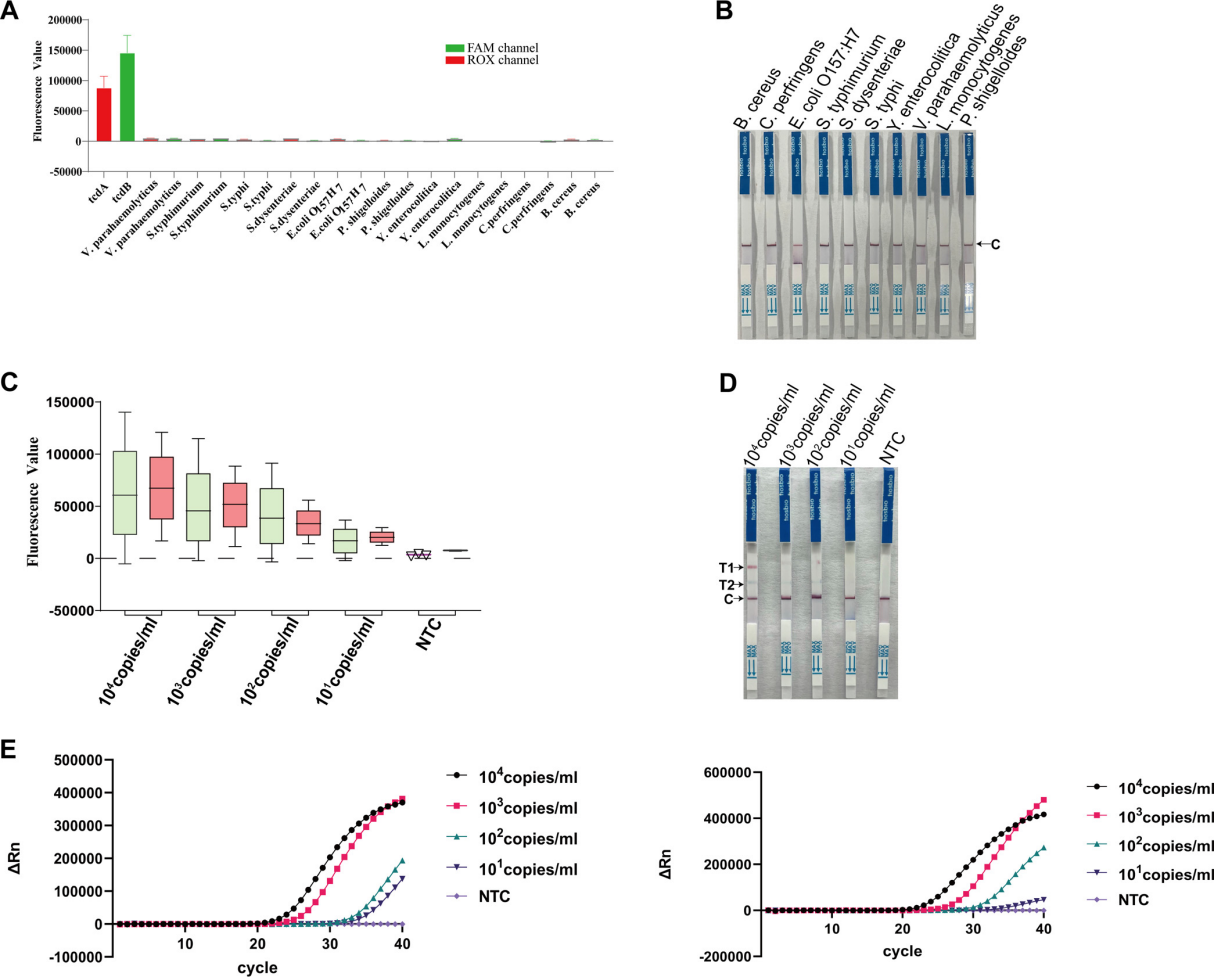
Validation of specificity and sensitivity. (A) Fluorescence signals of 10 major diarrhea-inducing pathogens. V. parahaemolyticus, Vibrio parahaemolyticus; *Salmonella typhimurium; Salmonella typhi*; *S. dysenteriae*, Shigella dysenteriae; *E. coli*, Escherichia coli; P. shigelloides, Plesiomonas shigelloides; Y. enterocolitica, Yersinia enterocolitica; L. monocytogenes, Listeria monocytogenes; *C. perfringens*, Clostridium perfringens; B. cereus, Bacillus cereus. (B) The test strip method was used to detect 10 major diarrhea-inducing pathogens. (C) Assessment of the sensitivity of fluorescence methods. (D) Assessment of the sensitivity of test strips. (E) Validation of the detection limits of commercially available qPCR reagents. The sensitivity for the *tcdA* gene is shown on the left, and the sensitivity for the *tcdB* gene is shown on the right.

To evaluate the specificity of the OC-MAB platform, DNAs extracted from 10 common diarrhea-causing pathogens were used as the templates. As delineated in Fig. 3A, only the FAM and ROX channels of the two toxin genes of C. difficile showed strong fluorescence signals, while the other 10 pathogenic bacteria showed no fluorescence signals. Similarly, when test strips were used to detect the DNA samples of 10 pathogenic bacteria, no color was formed in either detection line (Fig. 3B). Collectively, our results established that the OC-MAB platform had high specificity and no cross-reaction with other diarrhea-inducing pathogens.

A 10-fold gradient serial dilution of plasmid DNA containing the *tcdA* and *tcdB* genes was used as a template for sensitivity assessment. As shown in Fig. 3C, the detection limit for the *tcdA* and *tcdB* genes by the fluorescence method was 10^1^ copies/mL. Regarding the CRISPR dual-target test strip, the detection limit for the *tcdA* and *tcdB* genes was 10^2^ to 10^1^ copies/mL (Fig. 3D). In addition, the sensitivity in copies per milliliter of the purchased commercial C. difficile toxin gene fluorescence PCR assay kit was also investigated. The results showed that the sensitivity of the qPCR method was approximately 10^1^ copies/mL for both the *tcdA* and *tcdB* genes (Fig. 3E), suggesting that our platform was comparable or even superior to qPCR.

### Clinical sample detection performance using the OC-MAB platform.

**FIG 4:**
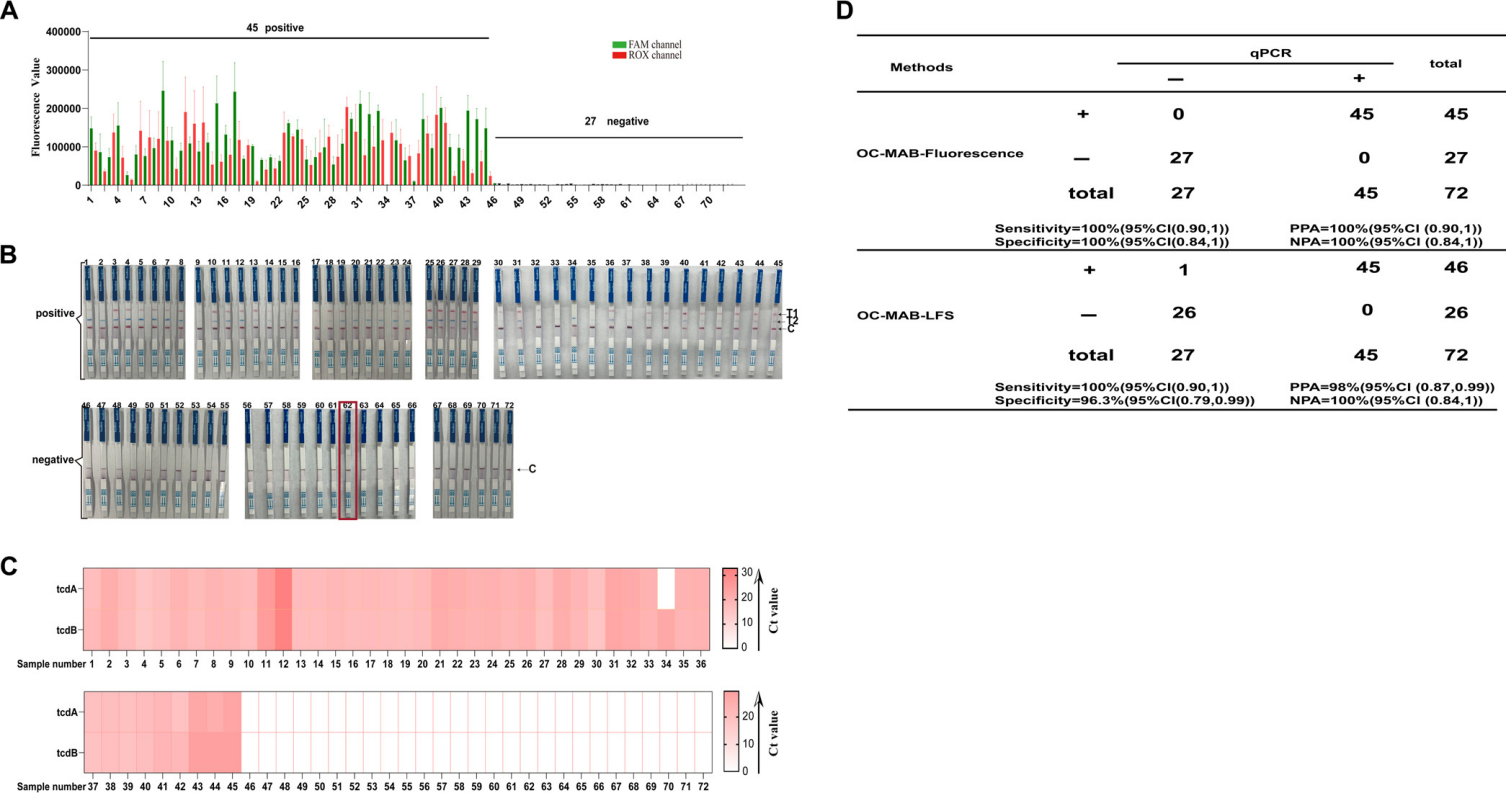
Evaluation of clinical samples. (A) Fluorescence signal diagram of 72 samples. (B) Diagram of test strip test results for 72 samples. (C) Results for 72 samples tested by the qPCR method. Ct, threshold cycle. (D) Consistency of the OC-MAB platform with the real-time fluorescence PCR method. CI, confidence interval; PPA, positive predictive value; NPA, negative predictive value; LFS, lateral flow strip.

Finally, the feasibility of the OC-MAB platform for the detection of the *tcdA* and *tcdB* toxin genes was evaluated using 72 clinical stool samples. More specifically, the samples were concurrently tested using qPCR and the OC-MAB platform. Our results showed that a total of 44 positive samples were positive for both *tcdA* and *tcdB*, with both the FAM channel and the ROX channel manifesting strong fluorescence signals, and 1 sample was positive for the *tcdB* gene, with the ROX channel showing a fluorescence signal. In contrast, 27 samples from cases with unexplained diarrhea showed no fluorescence signal (Fig. 4A). Again, the positive samples exerted a change in color on the test strip’s detection line (Fig. 4B). In contrast, there was one false-positive result for the negative samples using the test strips (Fig. 4B). Finally, in comparison, the single-tube method based on fluorescence readout achieved 100% consistency with qPCR. Conversely, the 2-step method based on test strip readout had a sensitivity of 100% and a specificity of 96.3% (Fig. 4D). The qPCR results for the 72 samples are portrayed in Fig. S5 and S6 in the supplemental material. Taken together, our results validate the robust clinical applicability of the OC-MAB platform.

## DISCUSSION

Clostridioides difficile infection (CDI) is a major concern in health care settings and is one of the leading causes of severe antibiotic-induced diarrhea worldwide. Accurate diagnosis of CDI is crucial, especially in the context of nosocomial surveillance, as the incidences of CDI-related morbidity, mortality, and even drug resistance in hospitalized patients have increased exponentially over the last 2 decades (29). Currently, the main pathogenic toxin factors of C. difficile are toxin A (TcdA), an enterotoxin, and toxin B (TcdB), a cytotoxin, which can cause diseases ranging from mild diarrhea to pseudomembranous colitis (30, 31). In addition, a more pathogenic type, 027/NAP1/BI, has been reported in Europe and the United States (32, 33). Currently, most health care providers rely on anaerobic cultures of C. difficile and related antigen tests such as the glutamate dehydrogenase (GDH) test. However, the main drawback of such tests is the inability to differentiate between virulent and nonvirulent strains. Moreover, their specificity needs to be improved (34, 35). In recent years, ongoing advancements in molecular biology techniques have resulted in the design of various PCR-based diagnostic methods for the detection of C. difficile, such as multiplex real-time fluorescence PCR (31, 36, 37). The most notable examples of such PCR-based diagnostic methods are the GeneXpert fluorescence quantification system and the Xpert C. difficile test developed by Cepheid. These methods detect *tcdB*, binary toxin (*cdtA* and *cdtB*), and *tcdC* with a deletion of nucleotide (nt) 117 and can determine whether a strain is an epidemic strain of type 027/NAP1/BI with high toxicity (38, 39). Although this system is highly sensitive and specific for the rapid and direct detection of C. difficile in fecal specimens, it is expensive and inconvenient. Therefore, this is a barrier to its use in resource-poor areas or communities. As a result, the development of a method that is comparable to qPCR is urgently needed. However, the method must be convenient and widely applicable, especially in resource-poor areas or communities.

This study is an upgrade of the previously established C. difficile CRISPR diagnostic platform (24) based on the orthogonal Cas12a and Cas13a dual systems combined with multiplex isothermal amplification technology. It is expected to be an ideal C. difficile diagnostic platform with the following unique advantages. First, through continuous optimization and adjustment, the orthogonal CRISPR dual system (Fig. 2D and E; see also Fig. S1 and S2 in the supplemental material) has been established. The CRISPR dual system, with the simultaneous detection of both genes, provides an improved detection efficiency. Furthermore, we used two types of result readouts, one of which relied on optical monitoring equipment such as a fluorescence PCR instrument. This readout method is the most appropriate for laboratories with sufficient resources and advanced capabilities. The other is a CRISPR dual-system test strip, suitable for rapid testing in low-resource areas or POCT sites. We created a highly efficient cutting system by changing the spiking system (Table S3), and the strips showed very strong bands. Moreover, the dual-system strips had a color change function, which allowed the reading of the results (Fig. S3 and S4). Finally, the lowest detection limit for the double-gene assay was as low as 10^2^ to 10^1^ copies/mL (Fig. 3C and D), demonstrating an exceptionally low sensitivity. At the same time, a comparison with the sensitivity of the qPCR method showed that our platform was comparable to qPCR (Fig. 3E). In addition, there was no cross-reactivity with several diarrheal pathogens (Fig. 3A and B), and the specificity was excellent. A total of 72 clinical samples were assessed methodologically, and the results showed reasonably good consistency with those of the qPCR method (Fig. 4). These results demonstrate that the platform has high accuracy and great potential for future application.

To the best of our knowledge, this study is the first report of an orthogonal CRISPR-based system for the detection of C. difficile toxin genes. However, this study also has some limitations. The *tcdA* and *tcdB* genes are not the only pathogenic genes of C. difficile. More comprehensive detection methods should be developed through epidemiological investigations of C. difficile. Therefore, in-depth research is needed to improve the functions of the platform and realize the full-coverage detection of *C. difficile*-related toxin genes.

## MATERIALS AND METHODS

### Multiple RPA response programs.

The total reaction mixture volume was reduced for the fluorescence-based single-tube orthogonal CRISPR detection platform to achieve optimal amplification. Briefly, 29.5 μL of a diluted reconstitution solution and 11.2 μL of nuclease-free water were added to TwistAmp tubes containing lyophilized enzyme components. Primers (T7-*tcdA*-F [5 μΜ], *tcdA*-R [5 μΜ], *tcdB*-F [5 μΜ], and *tcdB*-R [5 μΜ]) and template DNA were subsequently added to the tube. After mixing, 10 μL of the mixed solution was aliquoted into each PCR tube. Finally, 1 μL of 280 mM MgOAc was added to activate the reaction, and the mixture was immediately incubated at 37°C for 18 min.

The paraffin oil sealing method achieved orthogonal CRISPR detection using an immunochromatographic lateral flow strip. The reagents were mixed by the addition of 29.5 μL of a diluted rehydration solution, 11.2 μL of nuclease-free water, primers (T7-*tcdA*-F [10 μM], *tcdA*-R [10 μM], *tcdB*-F [10 μM], and *tcdB*-R [10 μM]), and the template DNA. After mixing, 2.5 μL of 280 mM magnesium acetate was finally added to activate the reaction, and 80 μL of paraffin oil was added to cover the surface, followed by incubation at 37°C for 18 min.

### Verification of LwaCas13a activity.

Briefly, the cleavage reaction was performed by mixing synthetic *tcdA* gene target RNA, 10× LwaCas13a buffer, LwaCas13a nuclease (1 μΜ), *tcdA*-crRNA (1 μΜ), and an ssRNA reporter (ROX-UUUUUU-BHQ2 [5 μM]). During incubation at 37°C for 30 min, the fluorescence of the ROX channel was monitored on a qPCR instrument in real time.

### Validation of LbCas12a and LwaCas13a cleavage preference.

Specifically, the reaction system of LbCas12a included dsDNA containing the *tcdB* gene, the Lachnospiraceae bacterium Cas12a(LbCas12a) protein (1 μM), *tcdB*-crRNA (1 μM), an ssDNA reporter (FAM-CCCCC-BHQ1 [1 μM]), an ssRNA reporter (ROX-UUUUUU-BHQ2 [5 μM]), and 10× NEBuffer 2.1 buffer. On the other hand, the reaction system of LwaCas13a included the dsDNA-containing *tcdA* gene, the LwaCas13a protein (50 nM), *tcdA*-crRNA (120 nM), an ssRNA reporter (ROX-UUUUUU-BHQ2 [5 μM]), an ssDNA reporter (FAM-CCCCC-BHQ1 [1 μM]), T7 RNA polymerase (50 U/μL), NTPMix (25 mM), a murine RNase inhibitor, and 10× NEBuffer 2.1. The fluorescence signals of the FAM and ROX channels were dynamically monitored every minute at 37°C using an ABI 7500 fluorescence quantitative real-time amplification system.

### Fluorescence-readout-based orthogonal CRISPR dual-system reaction.

The components of the orthogonal CRISPR dual-system assay included LbCas12a (200 nM), *tcdB*-crRNA (200 nM), LwaCas13a (50 nM), *tcdA*-crRNA (120 nM), a FAM-CCCCC-BHQ1 ssDNA reporter (1 μM), a ROX-UUUUUU-BHQ2 ssRNA reporter (5 μM), T7 RNA polymerase (5 U/μL), NTPMix (5 mM), a murine RNase inhibitor, and 10× NEBuffer 2.1. The CRISPR reagents were added to the PCR tube cap, while the multiplex RPA reagents were pipetted to the bottom of the PCR tube, and the tube was incubated at 37°C for 18 min. After a brief centrifugation step for 30 s, the CRISPR reagent was mixed with the multiplex RPA reagent, and the mixture was then incubated at 37°C for 30 min using an ABI 7500 qPCR instrument. The fluorescence signals of the FAM and ROX channels were simultaneously collected every minute.

### Orthogonal CRISPR dual-system reaction using a lateral flow strip.

First, LbCas12a (5 μM), *tcdB*-crRNA (1 μM), LwaCas13a (1 μM), *tcdA*-crRNA (10 μM), 10× NEBuffer 2.1, a FAM-TTTTTTTTTTT-biotin ssDNA reporter (5 μM), T7 RNA polymerase (50 U/μL), NTPMix (10 mM), and the amplicon amplified by multiplex RPA were added to PCR tubes. Subsequently, a DIG-UUUUUUUUUUUUUU-biotin ssRNA reporter (5 μM) and a murine RNase inhibitor were added to the reaction mixture, bringing the total volume to 30 μL with nuclease-free water. The reaction mixture was then incubated at 37°C for 20 min, and 20 μL of nuclease-free water was added to adjust the total volume to 50 μL. The CRISPR double-target test strip was inserted into the reaction mixture and incubated at room temperature for 3 to 5 min before the detection of the results.

### Sensitivity and specificity of the OC-MAB platform.

C. difficile-positive plasmids containing the *tcdA* and *tcdB* genes (initial copy number of 10^6^ copies/mL) were diluted in a 10-fold gradient. Next, the OC-MAB platform was used to evaluate the minimum detection limit. To confirm cross-reactivity, the specificity of the assay was evaluated using DNAs from various common intestinal diarrhea-inducing pathogens (see Table S2 in the supplemental material for information on specific strains).

### Application of the OC-MAB platform for clinical sample analysis.

A total of 45 stool samples from patients with C. difficile-positive anaerobic cultures (primary screening) were collected from several hospitals affiliated with Anhui Medical University. Anaerobic cultures were performed using anaerobic bags and gas-producing kits from bioMérieux and C. difficile chromogenic plates from Comagal. Incubation was carried out at 37°C for 48 h. Next, the strains were identified using matrix-assisted laser desorption ionization–time of flight mass spectrometry (MALDI-TOF MS). Moreover, 27 fecal samples from patients with diarrhea of an unknown origin were also collected from enteric diseases clinic. The inclusion criteria were (i) three or more episodes of diarrhea within 24 h and (ii) unformed stools (according to the Bristol classification). Meanwhile, fecal DNA was extracted using a fecal DNA extraction kit, followed by parallel detection using the OC-MAB platform and a C. difficile toxin gene detection kit (real-time fluorescence PCR). Presently, real-time fluorescence PCR is considered the gold standard for the detection of virulent C. difficile.

The qPCR procedure was performed according to the kit’s instructions, using an ABI 7500 instrument with a reaction mixture volume of 30 μL and a program of 40 cycles, as follows: 50°C for 2 min, 95°C for 5 min, 95°C for 15 s, and 55°C for 30 s. Finally, the fluorescence signals were analyzed. The FAM channel represented the *tcdA* gene, the Cy5 channel represented the *tcdB* gene, and the VIC channel represented the internal reference gene.

Finally, the performance of the OC-MAB platform was assessed by comparing its detection results to those obtained by qPCR.

## References

[1] Choi JR, Hu J, Tang R, Gong Y, Feng S, Ren H, Wen T, Li X, Wan Abas WAB, Pingguan-Murphy B, Xu F. 2016. An integrated paper-based sample-to-answer biosensor for nucleic acid testing at the point of care. Lab Chip 16:611–621. doi:10.1039/c5lc01388g. 26759062

[2] Singh C, Roy-Chowdhuri S. 2016. Quantitative real-time PCR: recent advances. Methods Mol Biol 1392:161–176. doi:10.1007/978-1-4939-3360-0_15. 26843055

[3] Subirats J, Royo E, Balcazar JL, Borrego CM. 2017. Real-time PCR assays for the detection and quantification of carbapenemase genes (blaKPC, blaNDM, and blaOXA-48) in environmental samples. Environ Sci Pollut Res Int 24:6710–6714. doi:10.1007/s11356-017-8426-6. 28084599

[4] Troxler S, Marek A, Prokofieva I, Bilic I, Hess M. 2011. TaqMan real-time reverse transcription-PCR assay for universal detection and quantification of avian hepatitis E virus from clinical samples in the presence of a heterologous internal control RNA. J Clin Microbiol 49:1339–1346. doi:10.1128/JCM.01626-10. 21307216 PMC3122850

[5] Zhang F, Wen Y, Guo X. 2014. CRISPR/Cas9 for genome editing: progress, implications and challenges. Hum Mol Genet 23:R40–R46. doi:10.1093/hmg/ddu125. 24651067

[6] Sakuma T, Yamamoto T. 2017. Magic wands of CRISPR—lots of choices for gene knock-in. Cell Biol Toxicol 33:501–505. doi:10.1007/s10565-017-9409-6. 28828704

[7] Hryhorowicz M, Lipiński D, Zeyland J, Słomski R. 2017. CRISPR/Cas9 immune system as a tool for genome engineering. Arch Immunol Ther Exp (Warsz) 65:233–240. doi:10.1007/s00005-016-0427-5. 27699445 PMC5434172

[8] Zetsche B, Gootenberg JS, Abudayyeh OO, Slaymaker IM, Makarova KS, Essletzbichler P, Volz SE, Joung J, van der Oost J, Regev A, Koonin EV, Zhang F. 2015. Cpf1 is a single RNA-guided endonuclease of a class 2 CRISPR-Cas system. Cell 163:759–771. doi:10.1016/j.cell.2015.09.038. 26422227 PMC4638220

[9] Chen JS, Ma E, Harrington LB, Da Costa M, Tian X, Palefsky JM, Doudna JA. 2018. CRISPR-Cas12a target binding unleashes indiscriminate single-stranded DNase activity. Science 360:436–439. doi:10.1126/science.aar6245. 29449511 PMC6628903

[10] Li S-Y, Cheng Q-X, Wang J-M, Li X-Y, Zhang Z-L, Gao S, Cao R-B, Zhao G-P, Wang J. 2018. CRISPR-Cas12a-assisted nucleic acid detection. Cell Discov 4:20. doi:10.1038/s41421-018-0028-z. 29707234 PMC5913299

[11] Abudayyeh OO, Gootenberg JS, Konermann S, Joung J, Slaymaker IM, Cox DBT, Shmakov S, Makarova KS, Semenova E, Minakhin L, Severinov K, Regev A, Lander ES, Koonin EV, Zhang F. 2016. C2c2 is a single-component programmable RNA-guided RNA-targeting CRISPR effector. Science 353:aaf5573. doi:10.1126/science.aaf5573. 27256883 PMC5127784

[12] East-Seletsky A, O’Connell MR, Knight SC, Burstein D, Cate JH, Tjian R, Doudna JA. 2016. Two distinct RNase activities of CRISPR-C2c2 enable guide-RNA processing and RNA detection. Nature 538:270–273. doi:10.1038/nature19802. 27669025 PMC5576363

[13] Gootenberg JS, Abudayyeh OO, Lee JW, Essletzbichler P, Dy AJ, Joung J, Verdine V, Donghia N, Daringer NM, Freije CA, Myhrvold C, Bhattacharyya RP, Livny J, Regev A, Koonin EV, Hung DT, Sabeti PC, Collins JJ, Zhang F. 2017. Nucleic acid detection with CRISPR-Cas13a/C2c2. Science 356:438–442. doi:10.1126/science.aam9321. 28408723 PMC5526198

[14] Guo L, Sun X, Wang X, Liang C, Jiang H, Gao Q, Dai M, Qu B, Fang S, Mao Y, Chen Y, Feng G, Gu Q, Wang RR, Zhou Q, Li W. 2020. SARS-CoV-2 detection with CRISPR diagnostics. Cell Discov 6:34. doi:10.1038/s41421-020-0174-y. 32435508 PMC7235268

[15] Arizti-Sanz J, Freije CA, Stanton AC, Petros BA, Boehm CK, Siddiqui S, Shaw BM, Adams G, Kosoko-Thoroddsen T-SF, Kemball ME, Uwanibe JN, Ajogbasile FV, Eromon PE, Gross R, Wronka L, Caviness K, Hensley LE, Bergman NH, MacInnis BL, Happi CT, Lemieux JE, Sabeti PC, Myhrvold C. 2020. Streamlined inactivation, amplification, and Cas13-based detection of SARS-CoV-2. Nat Commun 11:5921. doi:10.1038/s41467-020-19097-x. 33219225 PMC7680145

[16] Jiang Y, Hu M, Liu A-A, Lin Y, Liu L, Yu B, Zhou X, Pang D-W. 2021. Detection of SARS-CoV-2 by CRISPR/Cas12a-enhanced colorimetry. ACS Sens 6:1086–1093. doi:10.1021/acssensors.0c02365. 33683104

[17] Ding X, Yin K, Li Z, Lalla RV, Ballesteros E, Sfeir MM, Liu C. 2020. Ultrasensitive and visual detection of SARS-CoV-2 using all-in-one dual CRISPR-Cas12a assay. Nat Commun 11:4711. doi:10.1038/s41467-020-18575-6. 32948757 PMC7501862

[18] He C, Lin C, Mo G, Xi B, Li AA, Huang D, Wan Y, Chen F, Liang Y, Zuo Q, Xu W, Feng D, Zhang G, Han L, Ke C, Du H, Huang L. 2022. Rapid and accurate detection of SARS-CoV-2 mutations using a Cas12a-based sensing platform. Biosens Bioelectron 198:113857. doi:10.1016/j.bios.2021.113857. 34894625 PMC8635686

[19] Patchsung M, Jantarug K, Pattama A, Aphicho K, Suraritdechachai S, Meesawat P, Sappakhaw K, Leelahakorn N, Ruenkam T, Wongsatit T, Athipanyasilp N, Eiamthong B, Lakkanasirorat B, Phoodokmai T, Niljianskul N, Pakotiprapha D, Chanarat S, Homchan A, Tinikul R, Kamutira P, Phiwkaow K, Soithongcharoen S, Kantiwiriyawanitch C, Pongsupasa V, Trisrivirat D, Jaroensuk J, Wongnate T, Maenpuen S, Chaiyen P, Kamnerdnakta S, Swangsri J, Chuthapisith S, Sirivatanauksorn Y, Chaimayo C, Sutthent R, Kantakamalakul W, Joung J, Ladha A, Jin X, Gootenberg JS, Abudayyeh OO, Zhang F, Horthongkham N, Uttamapinant C. 2020. Clinical validation of a Cas13-based assay for the detection of SARS-CoV-2 RNA. Nat Biomed Eng 4:1140–1149. doi:10.1038/s41551-020-00603-x. 32848209

[20] Tao D, Liu J, Nie X, Xu B, Tran-Thi T-N, Niu L, Liu X, Ruan J, Lan X, Peng G, Sun L, Ma Y, Li X, Li C, Zhao S, Xie S. 2020. Application of CRISPR-Cas12a enhanced fluorescence assay coupled with nucleic acid amplification for the sensitive detection of African swine fever virus. ACS Synth Biol 9:2339–2350. doi:10.1021/acssynbio.0c00057. 32786346

[21] Wang X, Ji P, Fan H, Dang L, Wan W, Liu S, Li Y, Yu W, Li X, Ma X, Ma X, Zhao Q, Huang X, Liao M. 2020. CRISPR/Cas12a technology combined with immunochromatographic strips for portable detection of African swine fever virus. Commun Biol 3:62. doi:10.1038/s42003-020-0796-5. 32047240 PMC7012833

[22] Marques M-C, Sanchez-Vicente J, Ruiz R, Montagud-Martinez R, Marquez-Costa R, Gomez G, Carbonell A, Daros J-A, Rodrigo G. 2022. Diagnostics of infections produced by the plant viruses TMV, TEV, and PVX with CRISPR-Cas12 and CRISPR-Cas13. ACS Synth Biol 11:2384–2393. doi:10.1021/acssynbio.2c00090. 35793201 PMC9295153

[23] Wang Y, Ke Y, Liu W, Sun Y, Ding X. 2020. A one-pot toolbox based on Cas12a/crRNA enables rapid foodborne pathogen detection at attomolar level. ACS Sens 5:1427–1435. doi:10.1021/acssensors.0c00320. 32337966

[24] Jiang T, Hu X, Lin C, Xia Z, Yang W, Zhu Y, Xu H, Tang H, Shen J. 2023. Rapid visualization of *Clostridioides difficile* toxins A and B by multiplex RPA combined with CRISPR-Cas12a. Front Microbiol 14:1119395. doi:10.3389/fmicb.2023.1119395. 36970685 PMC10030577

[25] Gootenberg JS, Abudayyeh OO, Kellner MJ, Joung J, Collins JJ, Zhang F. 2018. Multiplexed and portable nucleic acid detection platform with Cas13, Cas12a, and Csm6. Science 360:439–444. doi:10.1126/science.aaq0179. 29449508 PMC5961727

[26] Tian T, Qiu Z, Jiang Y, Zhu D, Zhou X. 2022. Exploiting the orthogonal CRISPR-Cas12a/Cas13a trans-cleavage for dual-gene virus detection using a handheld device. Biosens Bioelectron 196:113701. doi:10.1016/j.bios.2021.113701. 34653714

[27] Li L, Duan C, Weng J, Qi X, Liu C, Li X, Zhu J, Xie C. 2022. A field-deployable method for single and multiplex detection of DNA or RNA from pathogens using Cas12 and Cas13. Sci China Life Sci 65:1456–1465. doi:10.1007/s11427-021-2028-x. 34962615 PMC8713540

[28] Mahas A, Marsic T, Lopez-Portillo Masson M, Wang Q, Aman R, Zheng C, Ali Z, Alsanea M, Al-Qahtani A, Ghanem B, Alhamlan F, Mahfouz M. 2022. Characterization of a thermostable Cas13 enzyme for one-pot detection of SARS-CoV-2. Proc Natl Acad Sci USA 119:e2118260119. doi:10.1073/pnas.2118260119. 35763567 PMC9282225

[29] Romero-Rodriguez A, Martinez de la Pena C, Troncoso-Cotal S, Guzman C, Sanchez S. 2022. Emerging alternatives against *Clostridioides difficile* infection. Anaerobe 78:102638. doi:10.1016/j.anaerobe.2022.102638. 36210608

[30] Di Bella S, Ascenzi P, Siarakas S, Petrosillo N, di Masi A. 2016. *Clostridium difficile* toxins A and B: insights into pathogenic properties and extraintestinal effects. Toxins (Basel) 8:134. doi:10.3390/toxins8050134. 27153087 PMC4885049

[31] Kim H, Jeong SH, Kim M, Lee Y, Lee K. 2012. Detection of *Clostridium difficile* toxin A/B genes by multiplex real-time PCR for the diagnosis of *C. difficile* infection. J Med Microbiol 61:274–277. doi:10.1099/jmm.0.035618-0. 21959205

[32] Novakova E, Stefkovicova M, Kopilec MG, Novak M, Kotlebova N, Kuijper E, Krutova M. 2020. The emergence of *Clostridium difficile* ribotypes 027 and 176 with a predominance of the *Clostridium difficile* ribotype 001 recognized in Slovakia following the European standardized *Clostridium difficile* infection surveillance of 2016. Int J Infect Dis 90:111–115. doi:10.1016/j.ijid.2019.10.038. 31707136 PMC6912155

[33] O’Connor JR, Johnson S, Gerding DN. 2009. *Clostridium difficile* infection caused by the epidemic BI/NAP1/027 strain. Gastroenterology 136:1913–1924. doi:10.1053/j.gastro.2009.02.073. 19457419

[34] Cheng J-W, Xiao M, Kudinha T, Xu Z-P, Sun L-Y, Hou X, Zhang L, Fan X, Kong F, Xu Y-C. 2015. The role of glutamate dehydrogenase (GDH) testing assay in the diagnosis of *Clostridium difficile* infections: a high sensitive screening test and an essential step in the proposed laboratory diagnosis workflow for developing countries like China. PLoS One 10:e0144604. doi:10.1371/journal.pone.0144604. 26659011 PMC4676637

[35] Chen S, Gu H, Sun C, Wang H, Wang J. 2017. Rapid detection of *Clostridium difficile* toxins and laboratory diagnosis of *Clostridium difficile* infections. Infection 45:255–262. doi:10.1007/s15010-016-0940-9. 27601055

[36] Huang H, Weintraub A, Fang H, Nord CE. 2009. Comparison of a commercial multiplex real-time PCR to the cell cytotoxicity neutralization assay for diagnosis of *Clostridium difficile* infections. J Clin Microbiol 47:3729–3731. doi:10.1128/JCM.01280-09. 19741082 PMC2772590

[37] Wroblewski D, Hannett GE, Bopp DJ, Dumyati GK, Halse TA, Dumas NB, Musser KA. 2009. Rapid molecular characterization of *Clostridium difficile* and assessment of populations of *C. difficile* in stool specimens. J Clin Microbiol 47:2142–2148. doi:10.1128/JCM.02498-08. 19403775 PMC2708487

[38] Babady NE, Stiles J, Ruggiero P, Khosa P, Huang D, Shuptar S, Kamboj M, Kiehn TE. 2010. Evaluation of the Cepheid Xpert *Clostridium difficile* Epi assay for diagnosis of *Clostridium difficile* infection and typing of the NAP1 strain at a cancer hospital. J Clin Microbiol 48:4519–4524. doi:10.1128/JCM.01648-10. 20943860 PMC3008447

[39] Shin S, Kim M, Kim M, Lim H, Kim H, Lee K, Chong Y. 2012. Evaluation of the Xpert *Clostridium difficile* assay for the diagnosis of *Clostridium difficile* infection. Ann Lab Med 32:355–358. doi:10.3343/alm.2012.32.5.355. 22950071 PMC3427823

