# Supplementary material for: Establishment of a Novel Detection Platform for Clostridioides difficile Toxin Genes Based on Orthogonal CRISPR
Source: Microbiol Spectr. 2023 Jun 28;11(4):e01886-23. doi: 10.1128/spectrum.01886-23 (PMC10434169; doi:10.1128/spectrum.01886-23)
Supplement: Supplemental file 1 — Supplemental material. Download spectrum.01886-23-s0001.pdf, PDF file, 2.8 MB [file spectrum.01886-23-s0001.pdf]

Supplementary Material

Supplementary Table 1. Sequence of primers/crRNAs/ssDNAs/ssRNAs/T7

Primers/crRNAs /ssDNAs/ssRNAs/T7	Sequence (5'-3')
T7-*tcdA*-RPA-F	GAAATTAATACGACTCACTATAGGGCATAGAGTCTGAT AATAACTTCACAGATACT
tcdA-RPA-R	ATTTATGAAATCATAGTAAGCTGACGCATA
tcdB-RPA-F	GAGAATCAATAAACTATACTGGTTGGTTAG
tcdB-RPA-R	CTATTCACTAATCACTAATTGAGCTGTATC
Cas13a-*tcdA*-crRNA	GAUUUAGACUACCCCAAAAACGAAGGGGACUAAAAC AGAGUUUUCUGCGGUAGCUGAAUUAAAU
Cas12a-*tcdB*-crRNA	UAAUUUCUACUAAGUGUAGAU CAGAUGAAUAUAUUGCAGCAA
ssDNA-FQ1	FAM-CCCCC-BHQ1
ssDNA-FB	FAM-TTTTTTTATTTTTTT-Biotin
ssRNA-RQ2	ROX-UUUUUU-BHQ2
ssRNA-DB	Dig-UUUUUUUUUUUU-Biotin
T7 promoter	GAAATTAATACGACTCACTATAGGG
tcdA target RNA	AACCTAGTAATAGAACAAGTAAAAAATAGATATCAATTT TTAAACCAACACCTTAACCCAGCCATAGAGTCTGATAAT AACTTCACAGATACTACTAAAATTTTTCATGATTCATTAT TTAATTCAGCTACCGCAGAAAACTCTATGTTTTTAACAA AAATAGCACCATACTTACAAGTAGGTTTTATGCCAGAAG CTCGCTCCACAATAAGTTTAAGTGGTCCAGGAGCTTATG CGTCAGCTTACTATGATTTCATAAATTTACAAGAAAATAC TAT

Supplementary Table 2. Bacterial strains involved in this study.

Bacteria	Source
C. difficile	clinical isolates
Bacillus cereus	CMCC (B)63301
Clostridium perfringens	ATCC 13124
Escherichia coli O157:H7	ATCC 700728
Salmonella typhimurium	clinical isolates
Shigella dysentery	CMCC (B) 51105
Salmonella typhi	clinical isolates
Yersinia enterocolitica	CMCC (B) 50024
Vibrio parahaemolyticus	ATCC 17802
Listeria monocytogenes	ATCC 19115
Plesiomonas shigelloides	ATCC 14029

Note: CMCC, China Medical Culture Collection; ATCC, American Type Culture Collection;

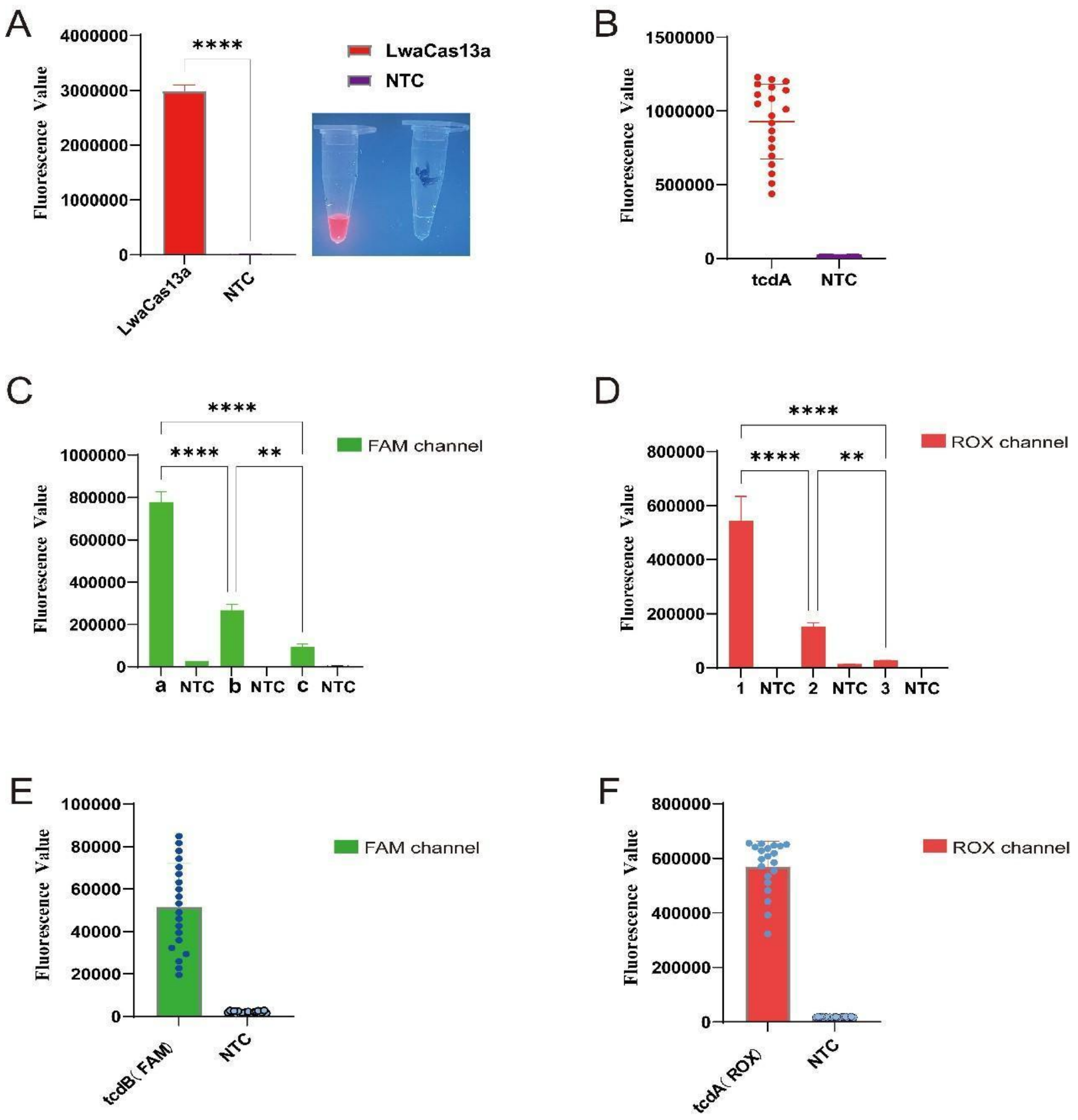

Supplementary Figure S1. Exploration of the dual systems. (A) Verification of Cas13a protein cleavage activity, the fluorescence was irradiated by a 365nm UV flashlight and photographed with a smartphone. NTC, no template control. Data are shown as mean ± SD (n=3). Statistical significance was calculated using two-tailed Student's test; ****, $p < 0.0001$. (B) Cleavage results of Cas13a targeting RPA product transcribed by T7. NTC, no template control. (C) Cas12a cleavage efficiency in different volumes. a, two-step method, i.e. RPA amplification (50 μL) was performed first, and then transfer the product to the Cas12a reaction system for detection of FAM. NTC, no template control. b,The RPA reaction mix enzyme component (dilute rehydrated solution + nuclease-free water + lyophilised enzyme) was pre-dissolved and mixed, followed by the addition of primers, DNA, etc. After mixing, 10 μL was taken as reaction reagent, followed by the addition of MgOAC, and the Cas12a reaction mix reagent was added to the cap of the tube. NTC, no template control. c, the total volume of RPA reaction and Cas12a reaction was 39.8 μL. NTC, no template control. Data are shown as mean ± SD (n=3). Statistical significance was calculated using two-tailed Student's test; **, $p < 0.01$; ****, $p < 0.0001$. (D) Cas13a

cleavage efficiency in different volumes. 1, two-step method, i.e. RPA amplification (50 μL) was performed first, and then transfer the product to the Cas12a reaction system for detection of ROX. NTC, no template control. 2, The RPA reaction mix enzyme component (dilute rehydrated solution + nuclease-free water + lyophilised enzyme) was pre-dissolved and mixed, followed by the addition of primers, DNA, etc. After mixing, 10 μL was taken as reaction reagent, followed by the addition of MgOAC, and the Cas13a reaction mix reagent was added to the cap of the tube. NTC, no template control. 3, the total volume of RPA reaction and Cas13a reaction was 41.8 μL. NTC, no template control. Data are shown as mean ± SD (n=3). Statistical significance was calculated using two-tailed Student's test; **, $p < 0.01$; ****, $p < 0.0001$. (E) Single-tube Cas12a cleavage reaction, NTC, no template control. Data are shown as mean ± SD (n=3). (F) Single-tube Cas13a cleavage reaction, NTC, negative control without template. Data are shown as mean ± SD (n=3).

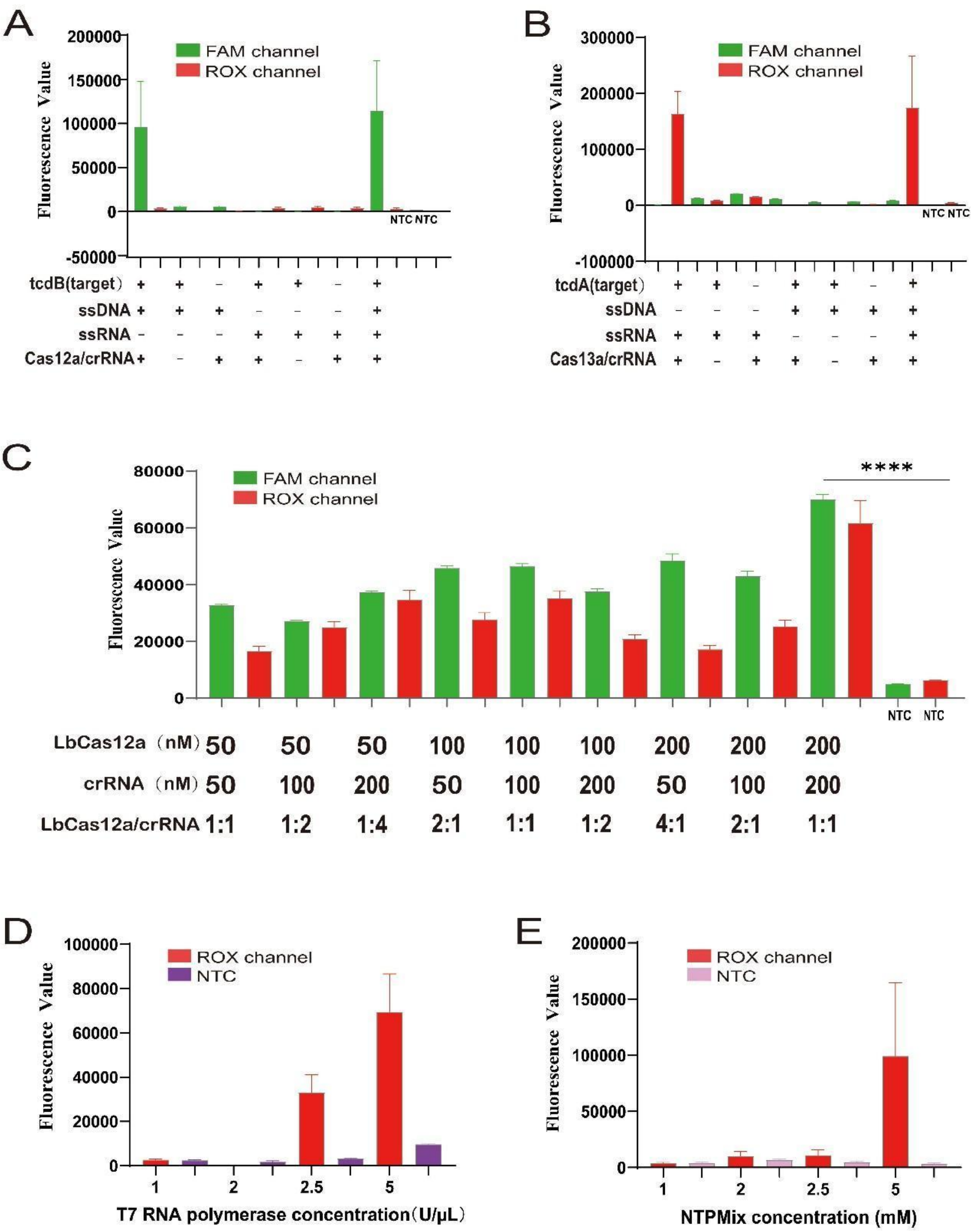

Supplementary Figure S2. Validation and optimization of the dual system. (A) Cas12a protein only cleaved ssDNA when ssDNA and ssRNA were coexisted in the system. (B) Cas13a protein only cleaved ssRNA when ssDNA and ssRNA were coexisted in the system. (C) Serial ratios of Cas12a:crRNA (*tcdB*) were tested with the Cas13a:crRNA (*tcdA*) concentration ratio fixed at 50nM:120nM. The optimal ratio was selected according to the fluorescence intensity. NTC: no template control. ****, $p < 0.0001$. (D) The optimal concentration of T7RNA polymerase was selected according to the fluorescence results of the ROX channel. NTC: no template control. (E) The optimal concentration of NTPMix was chosen according to the fluorescence signal of the ROX channel, NTC: no template control. All data were acquired from three independent experiments.

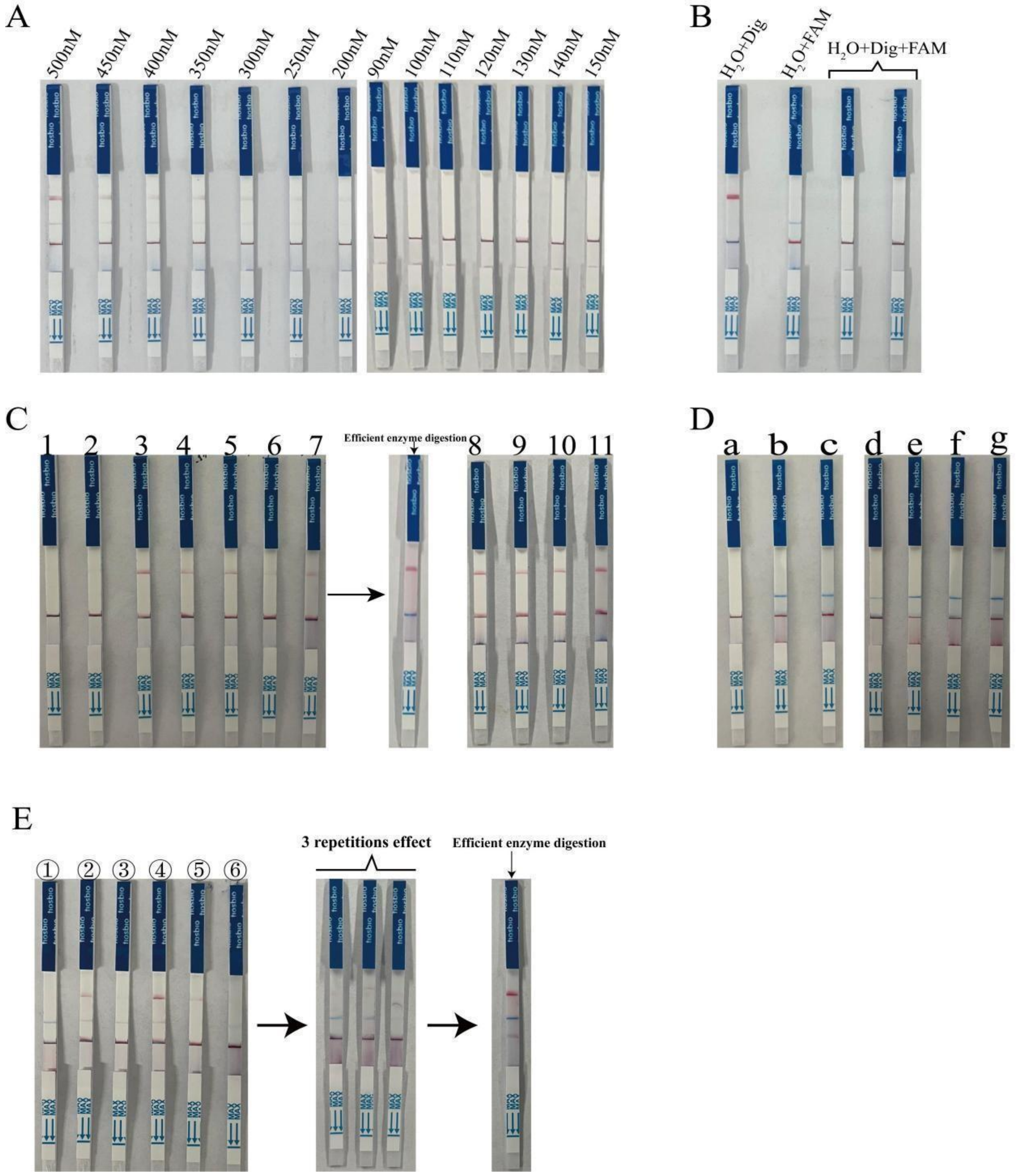

Supplementary Figure S3. High-efficiency CRISPR orthogonal dual system based on immunochromatography. (A) Optimization of probe concentrations. Probes with various concentrations were diluted in water with a total volume of 50 μL. Test strips were used to display immunochromatographical results. (B) Immunochromatographical results of system containing FAM probe, Dig probe, or both probes. (C) Exploration of high-efficiency system for Cas12a single-enzyme cleavage on test strips. 1, system containing 1 μM Cas12a and 1 μM *tcdB*-crRNA; 2, system containing 2 μM Cas12a and 1 μM *tcdB*-crRNA; 3, system containing 5 μM Cas12a, 1 μM *tcdB*-crRNA, and 1 μL Mg^{2+}; 4, system containing 5 μM Cas12a, 5 μM *tcdB*-crRNA; 5, system containing 5 μM Cas12a, 5 μM *tcdB*-crRNA, and 1 μL Mg^{2+}; 6, adding 1 μL of each of the probes to the system; 7, adding 1 μL of Dig-conjugated RNA probe and 0.5 μL of FAM-conjugated DNA probe to the system; 8, using HOLMES as the system buffer; 9, using NEBuffer™ 2.1 as the system buffer; 10, using

CutSmart™ as the system buffer; 11, using NEBuffer™ 2.1 and additional Mg^{2+} as the system buffer. (D) Exploration of high-efficiency system for Cas13a single-enzyme cleavage on test strips. a, system containing 1 μM Cas13a and 1 μM *tcdA*-crRNA; b, system containing 1 μM Cas13a and 10 μM *tcdA*-crRNA; c, system containing 5 μM Cas13a and 10 μM *tcdA*-crRNA; d, using HOLMES as the system buffer; e, using NEBuffer™ 2.1 as the system buffer; f, using CutSmart™ as the system buffer; g, using NEBuffer™ 2.1 and additional Mg^{2+} as the system buffer. (E) Exploration of dual-enzyme-cleavage and high-efficiency dual systems on test strips. ① indicates that the concentration of NTPMix solution in the system was 25 mM. ② indicates that the concentration of NTPMix solution in the system was 10 mM with a volume of 3 μL. ③ means that the volume of NTPMix (10mM) in the system was 4 μL. ④ means that additional Mg^{2+} was added to the system of ②. ⑤ indicates that the amount of Cas protein added was increased to 2 μL. ⑥ indicates that 4 μL of each of the 2 crRNAs was added to on the system of ⑤.

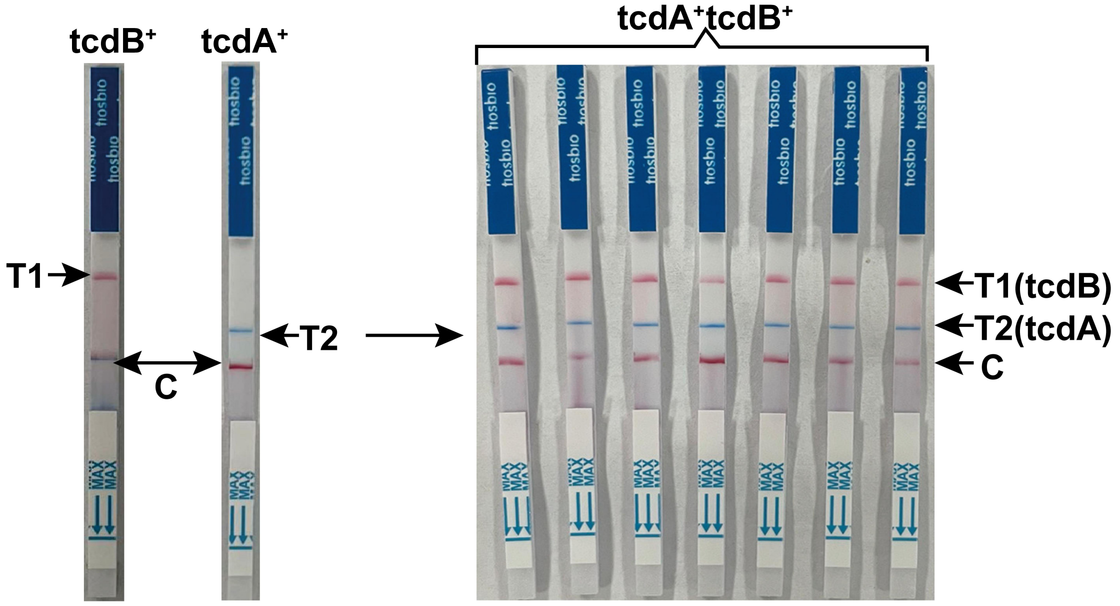

Supplementary Figure S4. Powerful colour change function of the strip after enzymatic cleavage. line T1 is Cas12a cleavage of FAM and Biotin labelled ssDNA probe, showing red. line T2 is Cas13a cleavage of Dig and Biotin labelled ssRNA probe, showing blue. line C is the quality control line, Cas12a single enzyme cleavage, showing blue; Cas13a single enzyme cleavage, showing red; Cas12a and Cas13a double enzyme cleavage, showing purple. Cas12a and Cas13a double cleaved, showing purple.

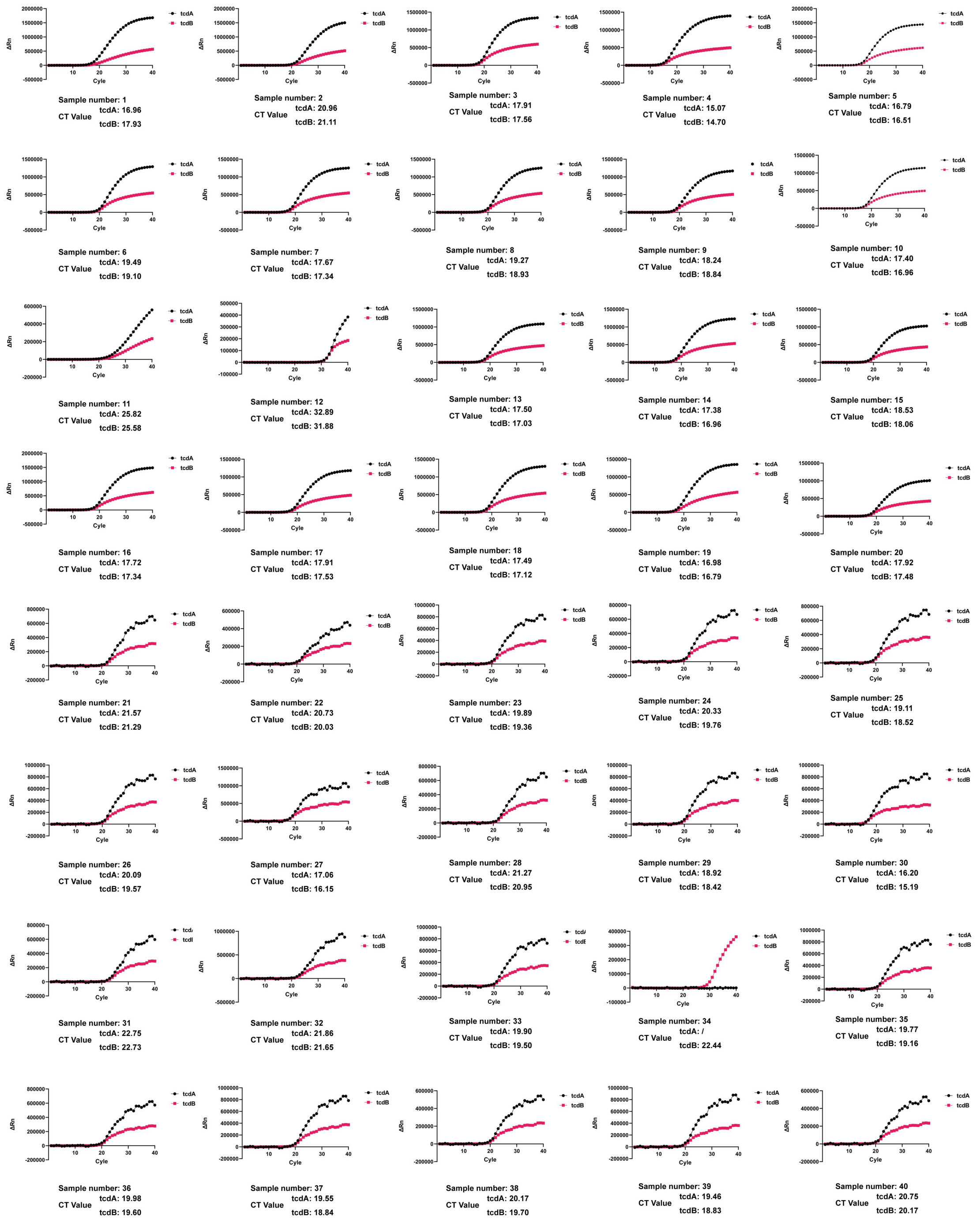

Supplementary Figure S5. qPCR results (standard curve and Ct values) for 45 positive samples.

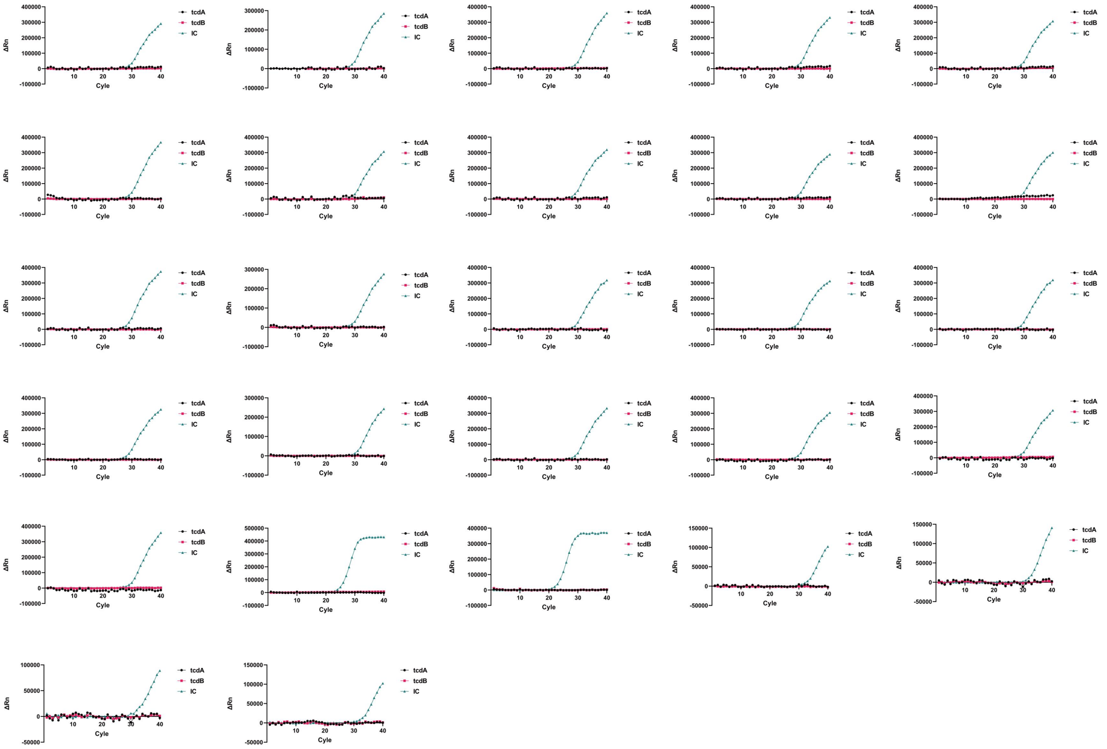

Supplementary Figure S6. qPCR results for 27 negative samples (standard curve). IC for Internal Control

Supplementary Table 3. CRISPR dual system efficient cutting system

Reagents	Amount to be added
Cas12a (5μM)	1μL
Cas13a (1μM)	1μL
tcdA-crRNA (10μM)	2μL
tcdB-crRNA (1μM)	2μL
FAM-7TA7T-Biotin (5μM)	0.5μL
Dig-12U-Biotin (5μM)	1μL
10×NEBuffer^{TM}2.1	3μL
Mg^{2+}	1μL
NTPMix (10mM)	3μL
T7 RNA polymerase (50 U/μL)	1μL
Murine RNase Inhibitor	0.5μL
RPA amplification products	5μL
H_2O	9μL
Total volume 30 μL	
